# Annotations

**Published:** 1895-08-03

**Authors:** 


### Annotations.

### Insanity in the Medical Profession.

It is not scientifically unwarrantable, or unreasonable from a common-sense point of view, to maintain that the doctor should be judged by the effect his physic has upon himself, and the parson by the character which his religion produces in himself. We ought to admit that an unhealthy medical man is an evidence of unsoundness either in the medical theories of the times or in the use to which he himself puts them. This is but fair, and if it be considered rather a transcendental position to take up at the present moment, one can hardly doubt that it will be regarded as a commonplace in the more cultured and rational times which lie in the womb of the centuries that are before us. Looking at certain aspects of medical insanity from the standpoint of high mental detachment, we cannot feel altogether happy at the figure cut by our profession in the lunacy returns of recent years. Lunacy varies with callings, as the recent and previous reports of the Commissioners amply prove. Theoretically there ought to be proportionately fewer lunatics among doctors than among any other class, because doctors know so well the causes and the means of prevention of lunacy. But what are the facts? The facts are that the medical profession ranks higher in the production of lunatics than all other classes except two. Costermongers and pedlars rank highest of all, and then follow woolstaplers and cloth merchants. The very next in order are physicians, surgeons, and general practitioners. The actual figures are even more striking than the bald statement of the fact. Thus, in every thousand costermongers and pedlars there were 20·1 lunatics in the quinquennium ending with 1893. In the same quinquennium there were 18·2 lunatics among woolstaplers and cloth merchants, and 15·8 among physicians, surgeons, and general practitioners. In marked contrast to these damaging figures are the figures relating to railway labourers, navvies, and miners. Those sturdy sons of toil only furnished 4·4 of their numbers to the ranks of lunacy in the quinquennium. We hold that as science advances, especially medical science, lunacy should diminish, not increase; and that the sanest and soundest of all classes should be doctors, whose business is "health." So far the facts are against us. But if we are to continue in the front ranks of practical science we must, without delay, find some means of so conducting our own profession as to at least preserve our sanity as well as our health and life. "Physician, heal thyself!" is a very significant dictum indeed.

### A Practical Aspect of Isolation.

In Dublin the question has been raised how far, in small-pox cases, isolation is to take the form of absolute exclusion of visitors, friends, relatives, clergymen, and so on, until the period of infection is quite passed. Speaking according to the strict letter of scientific demands there is no doubt that isolation should always be made absolutely complete. If we were dealing with the lower animals instead of with human beings we should make it complete; and if we did not, we should feel that we were lacking in thoroughness. But it is with things scientific as with things moral and things political where human beings are concerned; that is to say, the spirit of "compromise" must be admitted to our councils. What is to be remembered is that the one object of isolation is to prevent the spread of disease. If we attempt to make isolation absolutely complete without the most urgent necessity the certain result will be that hundreds of persons will resist, defy, and elude isolation in every possible way. The practical view to take is to consider in the first place the severity and extent of any given epidemic. In an epidemic neither severe nor extensive considerable latitude should be allowed in isolation. As the epidemic becomes more severe and more extensive so must isolation become stricter and more complete. In epidemics of the first magnitude separation should be absolute, and no doubt in such cases the terror of the public would make the most sympathetic submit to the manifest necessity. In cases of this kind "red tape" should as far as possible be kept out of sight, and good sense and good feeling everywhere have sway. It is an excellent rule for every official of every kind that he should always strive to do as he would be done by.

### Our Indian Troops.

Mr. Ernest Hart has made a forcible attack on the official heads of the Medical Department of our Indian Army, and if he is correct in his facts, it cannot be denied that the hardest things that he can say are fully justified. At immense expense a great army is maintained in India, and although all our "little wars" on the frontiers of that great country are attended with loss of life, the chief dangers to which our soldiers, whether native or European, are exposed in India, arise from disease and pestilence rather than from the chances of the battle-field. Of all countries in the world India is the place where the highest medical science and the most recent sanitary knowledge should be available for the preservation of our troops, and yet Mr. Hart tells us that "Indian sanitation is not a scientific but an official system. There does not exist there any independent medical opinion. All are in the employ and under the thumb of official authority. The oldest men in the service are the highest in official position. Their ignorance is often the standard by which their juniors are bound, their prejudices become a despotism and their follies a tyranny. It was for a long series of years as much as a man's promotion was worth to dare to avow the opinion that cholera was a waterborne disease, and that it was carried from place to place along the lines of human communication. . . . . And so it is that until lately Indian medical officers who dared to accept the patent facts, and to aver that particular outbreaks of cholera, of typhoid fever, and of dysentery were due to contamination of the drinking water, were subject to persecution. . . . Shameful to say, the Army Medical Regulations for the management of cholera are still produced and reprinted under the same death-dealing and ignorant superstition. All the most patent facts of our knowledge concerning cholera are still ignored, and in that scandalous public document, which is still supposed to be the handbook of our medical officers, cholera is treated as a contagious and mysterious disease, of which the origin is unknown, of which the very advent is to be the signal for panic and scare. . . . I cannot weary your patience by criticising in detail this most disgraceful document." Such assertions as these, made, as it has been, in an address to a large public meeting by one who is known to have given great attention to the subject and to have had recent opportunities for making inquiries on the spot, cannot be passed over. Either they must be proved to be without foundation or surely English public opinion will force on the Indian authorities a complete measure of reform.

